# Why is it Important to Assess and Treat Alexithymia in the Cardiologic Field? An Overview of the Literature

**DOI:** 10.2174/17450179-v19-230810-2022-HT15-4764-1

**Published:** 2023-08-15

**Authors:** Federica Sancassiani, Roberta Montisci, Luigi Meloni, Antonio Egidio Nardi, Mauro Giovanni Carta

**Affiliations:** 1 Department of Medical Sciences and Public Health, University of Cagliari, Asse Didattico E, SS 554 bivio Sestu 09042 Monserrato (CA), Italy; 2 Instituto de Psiquiatria (IPUB), Universidade Federal do Rio de Janeiro (UFRJ), Rio de Janeiro, RJ, Brazil

**Keywords:** Alexithymia, Cardiology, Assessment, Cardiovascular rehabilitation, Psychosomatics, Health psychology

## Abstract

**Background:**

Alexithymia has been found to be associated with several somatic illnesses, such as cardiovascular, indicating that it might be a risk factor for early death in the long-term course of post-myocardial infarction. From the cardiology perspective, the aim was to collect current evidence about the relationship between alexithymia and somatic illness.

**Methods:**

The literature was synthesized and summarized in a narrative format. The literature search was carried out in PubMed. Pertinent studies published in the last 50 years written in English were included and organized by three main topics (“The relation between alexithymia and somatic illness from the cardiology perspective”; “How do assess alexithymia?”; “Treating alexithymia”) to be discussed.

**Results:**

High alexithymia is a dimensional trait that affects around 10% of the general population and up to 55% of people with essential hypertension. Also, the link between alexithymia and cardiovascular activity has been pointed out. There are several validated tools to assess alexithymia, as well as treatment options.

**Conclusion:**

Knowledge about the main features of alexithymia, as well as its assessment and treatment, can promote a multifactorial approach to the primary, secondary, and tertiary prevention of cardiac diseases.

## 1 INTRODUCTION

At the beginning of ‘the 70s, the word “alexithymia” was proposed by Sifneos [1] from the ancient Greek “∂” (alpha privative), “lexis” (word), “thymos” (emotion), so it means “lack of words for emotions”. Originally, it was coined to describe some clinical features observed among people with psychosomatic disorders who had difficulty engaging in insight-oriented psychotherapy [2].

People with high alexithymia show deficiencies in emotional awareness and communication and have poor insight into their feelings, symptoms, and motivation. When asked about their feelings in emotional situations, they might experience confusion (*i.e*.: “I don’t know what is happening”), give a vague response (*i.e*.: “I do not feel fine”), mainly report bodily states (*i.e*.: “my stomach hurts”, “my legs are heavy”), or talk about behavior (*i.e*.: “I’d like to scream”, “I want to punch the wall”).

Due to these clinical characteristics, alexithymia is a construct referring to a personality trait characterized by: a) difficulty in discriminating one emotion from the others, as well as between somatic states and emotions; b) difficulty in communicating emotions to other people; c) lack of imaginative processes; d) cognitive style environmental stimulus oriented [1, 3-5].

Individuals with high alexithymia do not repress or hinder or deny emotions: they do not recognize them, lacking mental representations of emotions due to a deficit in their cognitive processing [6-8]. This deficit leads to an impairment in understanding and regulating emotions by communicating them [9]. Alexithymia is characterized by amplifying the somatic sensations associated with emotional arousal, or by misinterpreting these as symptoms of disease [10]. Furthermore, alexithymia may affect illness behavior through social mechanisms that lead to somatic symptom presentation [11]. People with a high level of alexithymia perceive less social support, have fewer close relationships and have poor social skills, such that alexithymia is linked with reduced social support or impaired relationships [11, 12].

Alexithymia is a dimensional trait that is thought to affect 10% of the general population when it is relevant clinically [13, 14]. The research pointed out the presence of “alexithymic mental areas”, indicating that it does not refer to an absolute inability to feel and state emotions. There also could be a “secondary alexithymia” after transplant, dialysis, intensive care [15], or after trauma [16] that implies a fast regression of effects to a pre-conceptual level, without any ability to differentiate them. In these cases, alexithymia seems a defense mechanism more than a primary deficit.

William Shakespeare, in his play *Macbeth*, wrote “Give sorrow words; the grief that does not speak knits up the o-er wrought heart and bids it breaks”. This sentence seems to describe what happens to people with a high level of alexithymia. They suffer from somatic symptoms more than psychological or relational difficulties. They can show sudden anger or cry without knowing why; they tend to express emotions through action and behaviors; they show a poor oneiric life. Furthermore, they appear as “pseudo-normal” persons by their behaviors, with poor gesturing and empathy. People with high alexithymia often show an undifferentiated negative emotive state that often makes them bore to the interlocutor. During psychodynamic psychotherapy, such people have been described as unproductive, unimaginative, boring, and stiff. Therapists have high difficulty establishing therapeutic relationships with them, and such psychotherapies lead to poor benefit [2, 9, 17].

The alexithymic deficit in processing feelings seems to impact mental and somatic health [2] through behaviors acted as a way to regulate affective activation (*i.e*.: eating disorders, alcohol abuse), psychopathology associated with emotional dysregulation (*i.e*.: panic disorder, somatoform disorder), the post-traumatic shutdown of emotions (*i.e*.: post-traumatic stress disorder, acute reactions to severe organic diseases), altered autonomic, immune and endocrine response (*i.e*.: vulnerability to inflammatory processes), somatosensory amplification (*i.e*.: pain), and dysfunctional healthcare-seeking behavior, during acute myocardial infarction [18-21].

Alexithymia has been found to be associated with cardiovascular diseases and predicts cardiovascular risk in healthy people [22]. Furthermore, alexithymia is a risk factor for early death in the long-term course of post-myocardial infarction, even if the underlying mechanisms are still unresolved [23-25].

Given the prevalence of alexithymia, the impact that it has in terms of somatic complaints, and its role as a risk factor in cardiovascular health [26], this overview aims to collect pertinent evidence about the relationship between alexithymia and somatic illness from the cardiology perspective, with particular attention to the assessment of alexithymia and its treatment, to provide a comprehensive framework about a multifactorial approach to the primary, secondary and tertiary prevention of cardiac diseases.

## 2 METHODS

The literature search was carried out in October 2022 in the electronic databases PubMed/Medline, by title/abstract, with the following keywords: “care-seeking behavior” OR “cardiovascular disease” OR “cardiovascular diseases” OR “cardiovascular health” OR “cardiovascular” OR “acute myocardial infarction” OR “somatic illness” OR “somatic disease” OR “hypertension” OR “interview” OR “questionnaire” OR “treatment” OR “psychotherapy” OR “psychological support” OR “death risk” AND “alexithymia.

All kinds of pertinent articles that were published in the last 50 years and written in English were included. It was decided to extend the search within the last 50 years because the initial papers related to alexithymia date back to that period. Relevant papers were also extracted by clicking “Related Articles” on a pertinent citation shown in PubMed, and from the references provided in the collected papers.

The pertinence and the relevance of the papers needed for their selection were judged by two of the authors (F.S. and M.G.C.).

Finally, the findings of relevant articles were reported and discussed in paragraphs focused on three main topics and summarized in Table **1**.

## 3 RESULTS

The search by combinations of the keywords revealed 1.803 articles. After the removal of duplicate articles, the pertinence of the remaining articles was analyzed.

Twenty-eight articles were included and then organized by three topics (“The relation between alexithymia and somatic illness from the cardiology perspective”; “How to assess alexithymia?”; “Treating alexithymia”) to be presented and discussed. As shown in Table **1**, we included 14 articles in Topic 1, 11 in Topic 2, and 3 in Topic 3.

### 3.1 Topic 1 - The Relation between Alexithymia and Somatic Illness from the Cardiology Perspective

The link between alexithymia and cardiovascular activity has been pointed out by research. Several studies show that alexithymia is related to higher levels of resting (tonic) sympathetic or cardiovascular activity and arousal, heart rate, and blood pressure reactivity to experimental stressors [2].

Coherently with these results, alexithymia was significantly high in samples of newly diagnosed yet untreated hypertensive subjects [27] and among treated hypertensive subjects [28]. A further study pointed out a prevalence of alexithymia of up to 55% in patients with essential hypertension, hypothesizing that people with alexithymia are vulnerable to states of heightened sympathetic arousal that lead to the development of essential hypertension [29].

In 2010, a 20 years-longitudinal study [24] on 2.321 Finnish men exploring the associations between baseline alexithymia and cardiovascular disease (CVD) showed that, after adjustments for age and several behavioral (smoking, alcohol consumption, physical activity), physiological (low- and high-density lipoprotein cholesterol, body mass index, systolic blood pressure, history of CVD), and psychosocial (marital status, education, depression) factors, the risk of CVD death was increased by 1.2%, for each 1-point increase in Toronto Alexithymia Scale-26 scores. This study pointed out that alexithymia is associated with increased cardiovascular mortality.

More recently, a longitudinal study on 1.122 young adults and adolescents of the same Finnish cohort [25] evaluated the association of cardiovascular health in adolescence and young adulthood with alexithymia 25 years later. The Authors used seven ideal cardiovascular health metrics (ICH index) including blood pressure, cholesterol and glucose levels, smoking, physical activity, body mass index, and diet. Adjusting for depression, age, social and lifestyle factors, they found that the ICH index was significantly associated with the TAS-20 total score, as well as both with Difficulty Identifying Feelings (DIF) and Difficulty Describing Feelings (DDF), so that poor cardiovascular health was associated with higher alexithymia scores.

Finally, in a study among a cohort of 83 people with ST-segment elevation myocardial infarction (STEMI), it was found that between individuals evaluated about their state in life and those who were not high alexithymia, was a significant determinant of early death in the long term (10 years later) after STEMI, controlling for age, sex and alexithymia level [23].

People with post-acute myocardial infarction (AMI) developed high levels of alexithymia within 3 to 6 months after discharge, with low temporal stability suggesting that secondary alexithymia could raise after AMI [30]. Patients with a previous AMI or established coronary heart disease (CHD) were found to delay responding to their symptoms more than patients with a first AMI. This delay time may be due to secondary alexithymia, resulting from previous cardiac events [30].

Other studies, among which a systematic review [21] and a meta-analysis [19], assessed people after they were discharged from the intensive unit just after AMI. These studies [18, 20, 31-33] pointed out that people who delayed longer to seek care have shown a significantly higher level of alexithymia suggesting that alexithymia is a relatively stable personality trait that may lead to dysfunctional health care-seeking behavior.

### 3.2 Topic 2 - How Do assess Alexithymia?

During the past years, different tools have been developed to assess alexithymia, including self-report questionnaires, observer-rated measures, structured interviews, and performance-based tests [34].

In the research field, the widest instrument used to assess alexithymia is the Toronto Alexithymia Scale – 20 item (TAS-20) [35, 36], which has been translated into several languages and validated in diverse cultural groups [37].

It is a self-report questionnaire that includes 20 items. The subject has to respond on a Likert scale composed of 5 points. The factorial analyses of the instrument point out three factors, that correspond to the main features of the construct: 1) difficulty in identifying emotions and distinguishing them from emotions and bodily sensations (DIF); 2) difficulty in describing and communicating emotions (DDF); 3) externally oriented thinking, that is too realistic, with poor fantasies (EOT). Timing to fill out the questionnaire can vary between 5-10 minutes. Four scores can be obtained from the questionnaire: one for each factor and one total score. The distribution in the general population is normal. Even if alexithymia should be considered a continuous trait as said above, the research pointed out that a score ≥ 61 indicates high alexithymia, a score <51 indicates low alexithymia, with an intermediate score indicating a medium level of alexithymia.

Before this version, the Toronto Alexithymia Scale (TAS) [38] was developed and used. The main difference with the TAS-20 is that the TAS includes 26 items, so it was replaced with the TAS-20, which is shorter to administer.

A critical issue that has been pointed out about the assessment of alexithymia by the TAS-20 is whether individuals with high levels of alexithymia can reliably self-evaluate their ability to identify and describe their own emotions [39]: there is the conceptual conundrum about reporting characteristics that, by definition, involve impaired introspection, thus raising questions about the validity of this approach, especially to capture the high end of alexithymia [2].

Another limit regards an overlap with self-report measures of negative affect, and the absence of items to assess fantasy and imaginal activities [35, 36, 40].

To partially address these issues, there are three interview-based alexithymia assessment approaches.

Several decades ago, Sifneos [1] was the first who recognized the need to assess alexithymia reliably and developed the Beth Israel Hospital Questionnaire (BIQ). This instrument includes 17 items rated dichotomously by a clinician. This tool was modified into a 12-item rating scale that assesses the lack of emotional awareness and the tendency for operational thinking, capturing the original, clinically based manifestations of alexithymia [35, 36].

Even if the BIQ was often used in the original or modified form as the criterion against which other alexithymia measures have been validated [2], it is not widely used due to the challenges of training to administer it, the time needed to complete the interview, the lack of standardized roles to obtain the information and, consequently, difficulties for good inter-rater reliability.

To overcome some of these limits, a more structured interview method for assessing alexithymia — the Toronto Structured Interview for Alexithymia (TSIA) was developed [41]. The TSIA includes 24 questions, six for each of the four main dimensions of the alexithymia construct: difficulty identifying feelings (DIF), difficulty describing feelings (DDF), an externally oriented style of thinking (EOT), and imaginal processes (IMP). The respondents are asked to provide illustrative self-reported examples that support their responses and are rated by a systematic probing, with each response coded on an anchored rating scale.

One potential constraint in TSIA usage is the amount of time it takes to administer and score, which is around 50 to 60 minutes. As such, a short version of the interview retaining item providing maximum information was tested [42]. It results in the elimination of 12 items and, despite the 50% reduction in the number of items, 65.22% of the information was retained, maintaining adequate accuracy, and will save approximately 25–30 minutes of administration and scoring time.

Another interview-based tool to assess alexithymia is the structured interview based on the Diagnostic Criteria for Psychosomatic Research (DCPR) [43], which consists of a series of psychosomatic syndromes, including alexithymia. The interview includes 59 items scored in a yes/no answer format. The cluster for alexithymia consists of 6 items, at least 3 of which are necessary to establish high levels of alexithymia. Two questions regard the ability to verbalize and communicate emotions, two questions concern cognitive features, such as the reduced ability to fantasize and external-related thinking, one question regards the presentation of somatic difficulties after the experience of strong emotions, and one last question concerns non-mentalized outbursts.

The item/questions regarding alexithymia are the following [44]:

1. When you experience something good or bad, are you able to describe your emotions (delight, joy, worry, sadness, anger)?

2. When you experience either good or bad events, do you talk about what has happened and what you feel inside of you?

3. Do you often daydream and let your imagination run away?

4. Do your thoughts concern your internal emotions and feelings more often?

5. When you experience a strong emotion, do you also feel physical reactions? (*i.e*.: sick to stomach, *etc.*?)

6. Have you ever had occasional but violent outbursts of anger, crying, or joy, that are inappropriate either in relationship with what was happening or your usual behavior?

Research has shown excellent inter-rater reliability [45], and a good correlation with self-reported measures of alexithymia [46].

The development of these structured interviews partially overcomes the concern about the reliability or validity of self-report assessments of alexithymia.

More Recently, the Toronto Alexithymia Scale – Informant Form (TAS-20-IF) version was developed [47], modifying the 20 items of the self-report TAS-20 by writing them in the third person (he/she/it).

The participants served as “informants” (*i.e*.: close friend, romantic partner, spouse, sibling, parent, adult child, *etc.*) for a “target” person who was selected among someone they knew very well.

As for the self-report version of the TAS-20, the TAS-20-IF maintains a five-point Likert response format, from 1 (strongly disagree) to 5 (strongly agree). Scoring is the same with higher scores indicating a greater degree of alexithymia. The validation study confirmed the three-factor structure of the TAS-20, as well the correlation between the two versions was statistically significant [47].

### 3.3 Topic 3 - Treating Alexithymia

People with a high level of alexithymia often experience psychological distress as somatic symptoms or by “emotional storms” [9] which cannot be interpreted in a meaningful way. These persons usually prefer a more somatic causal model compared to psychosocial explanations for their difficulties. People with high levels of alexithymia know that they do not feel good, but do not know how to say or describe what they are feeling. They have difficulty presenting material spontaneously and fixate on their physical symptoms and minute details of external events [9, 48]. Furthermore, the attitudes of people with high alexithymia towards psychotherapy and their expectations for the treatment outcome are limited, which may reduce their motivation [49].

Psychotherapy assumes that individuals have some access to their feelings. Thus, people with high alexithymia, who are unable to identify, differentiate, and articulate their emotions, often are a difficult challenge for a therapist. Alexithymia has little effect on treatment preferences, yet there was some tendency for people with alexithymia to prefer group therapy [48]. However, alexithymia was associated with poor outcomes in traditional psychodynamic psychotherapy [9, 48]. In the context of individual or group therapy, higher alexithymia elicited negative reactions from one's therapist such as frustration, helplessness, and anger, which partially contributed to the poor outcome. These negative reactions that therapists had during treatment with people with high alexithymia seem to be in response to the lack of positive emotion expressed by these patients, as well as to their impossibility to access emotions to give meaning to their difficulties [9, 48].

Supportive psychotherapy, including an initial part of psychoeducation, different from the traditional interpretative psychodynamic approach, could be more useful with people with high alexithymia [9]. In the beginning, the psychotherapist could explain to the patient that he/she usually undertakes emotions by their physical component (arousal) more than as feelings. Secondly, it could be useful to teach the patient that emotions have a limited lasting and intensity, as well as direct the attention of the patients to their behavioral component of emotions, such as sighs, gesturing, and movements, to start working on differentiating their emotions. Furthermore, supportive psychotherapy implies teaching the patients that emotions are not states that need to be repressed, denied, or discharged by action, but they are information useful to manage stressful situations as well as interpersonal relationships. Finally, it could be useful to teach the patient to pay more attention to their dream life, to amply the possibility to access their feelings.

Biofeedback, relaxation techniques, autogenic training, and meditative practices are therapeutic possibilities useful to start from the bodily component of emotions which is what people with high levels of alexithymia can access first [9, 49].

Another element that could be useful for the treatment of people with high alexithymia is building a “real relationship” with the patient [49], where the therapist shears his/her own feelings, fantasies, humor, and other imaginative activities during the therapeutic sessions by using emotionally balanced language to increase the patient’s lexicon for this kind of language. For people with high alexithymia, an honest expression of curiosity, emotional availability, and a reflective stance by the therapist are powerful resources which improve their deficit in recognizing emotions.

If individual sessions are useful to educate people with high alexithymia about their deficit in emotion regulation, the group setting allows them to improve their ability to recognize emotion through interaction with other people, especially about the impact of the poor empathy they usually show.

A study to evaluate the efficacy of group psychotherapy on 20 people who survived after an acute myocardial infarction showed a significant improvement in alexithymia after 4 months with one session/week, each lasting 90 minutes compared with an educational treatment focused on cardiovascular risk factors [50]. The group psychotherapy included several techniques, such as Jacobson’s progressive relaxation, and role-playing focused on communicating emotions in a nonverbal way, describing their own dreams to others verbally or in written form, and painting their own interior feelings. Patients were further asked to apply these techniques in their daily life.

The evaluation by the TAS was conducted before and after treatment. Furthermore, it was repeated by follow-up 6, 12, and 24 months after the end of the treatment. Over time, cardiac conditions were assessed in terms of the occurrence of new myocardial infarction, death, arrhythmia, the occurrence of angina, and hospitalization after angina. The results were very interesting, even if the small sample size: in comparison with the control group, TAS scores were significantly lower after the group therapy and remained lower during all the follow-up assessments. Furthermore, people who improved in alexithymia scores showed a frequency of cardiac complications significantly lower over time.

## CONCLUSION

This overview provides a synthesis of previously published studies regarding the relationship between alexithymia and somatic illness from the cardiology perspective. It points out the link between alexithymia and cardiovascular activity, the role of alexithymia as a risk factor for early death in the long-term course of post-myocardial infarction, as well as its impact on dysfunctional healthcare-seeking behavior when an acute myocardial infarction occurs. Finally, it describes validated assessment tools for the evaluation of alexithymia, as well as psychotherapeutic treatment options and implications.

Alexithymia is a personality trait that refers to difficulty identifying feelings, difficulty describing feelings to other people, a reduced ability to create fantasies, and a thought content that reveals a preoccupation with the minute details of bodily sensations and/or the external environment. These features reflect a deficit in the cognitive processing and regulation of emotions.

Alexithymia can be reliably assessed by several instruments, and it can be improved after supportive psychotherapeutic interventions able to impact the cognitive deficit to recognize emotions.

Literature suggested that a multifactorial approach to the primary, secondary, and tertiary prevention of cardiac diseases is needed to further reduce morbidity and mortality rates. While it remains unclear how alexithymia can contribute directly to organic diseases, such as cardiovascular ones, there is evidence that it is associated with several illnesses that involve disturbances in emotional regulation and that it may also negatively impact health behaviors.

Even if exhaustive, this overview does not systematically include all the literature in this field. However, its narrative approach allows clinicians to get basic knowledge about the features of the alexithymia construct and its prevalence among people with cardiovascular diseases, indicating that poor cardiovascular health is often associated with higher alexithymia. It also makes available some tips to evaluate alexithymia levels with valid instruments already used in the cardiologic field, as well as to indicate proper interventions to support patients with high levels of alexithymia to improve their ability to recognize emotions.

## Figures and Tables

**Table 1:** Main findings of the included studies.

**Topic**	**Authors [Reference]**	**Main Results**
***Topic 1 - The relation between alexithymia and somatic illness from the cardiology perspective.***	Jula *et al*, 1999 [27]	Alexithymia was significantly high in a sample of 237 people of both genders, 35-54 years older, newly diagnosed yet untreated hypertensive subjects, compared to an age- and gender-stratified random population sample of 146 normotensives. The association between high alexithymia and elevated blood pressure was significant independently of sodium and alcohol intake, body mass index, and physical fitness.
	Casagrande *et al*, 2019 [28]	Alexithymia was significantly higher among 810 hypertensive subjects than 431 normotensives. According to the presence of pharmacological treatment, treated hypertensive patients are more alexithymic than normotensive and not treated hypertensive patients. Considering the blood pressure control associated with the drug -therapy, people with uncontrolled hypertension are more alexithymic than normotensive and untreated hypertensive people.
	Todarello *et al*, 1995 [29]	Among 114 hypertensive patients, the prevalence of alexithymia was found up to 55%. The Authors hypothesized that people with alexithymia are vulnerable to states of heightened sympathetic arousal that lead to the development of essential hypertension.
	Toulmen *et al*, 2010 [24]	20 years-longitudinal study on 2.321 Finnish men exploring the associations between baseline alexithymia and CVD showed that, after adjustments for age and several behavioral (smoking, alcohol consumption, physical activity), physiological (low- and high-density lipoprotein cholesterol, body mass index, systolic blood pressure, history of CVD), and psychosocial (marital status, education, depression) factors, the risk of CVD death was increased by 1.2% for each 1-point increase in Toronto Alexithymia Scale-26 scores. This study pointed out that alexithymia is associated with increased cardiovascular mortality.
	Karukivi *et al*, 2020 [25]	A longitudinal study on 1.122 Finnish young adults and adolescents with alexithymia evaluated the association of their cardiovascular health 25 years later. The Authors used seven ideal cardiovascular health metrics (ICH index) including blood pressure, cholesterol and glucose levels, smoking, physical activity, body mass index, and diet. Adjusting for depression, age, and social and lifestyle factors, they found that less ideal cardiovascular health was associated with higher alexithymia scores.
	Carta *et al*, 2022 [23]	A study among a cohort of 83 people STEMI pointed out that, between individuals evaluated about their state in life and those who were not, high alexithymia was a significant determinant of early death in the long term (10 years later) after STEMI, controlling for age, sex and alexithymia level.
	Kojimaa *et al*, 2001 [24]	People with post-AMI developed high levels of alexithymia within 3 to 6 months after discharge, with low temporal stability suggesting that secondary alexithymia could raise after AMI. Patients with a previous AMI or established CHD were found to delay responding to their symptoms more than patients with a first AMI. This delay time may be due to secondary alexithymia, resulting from previous cardiac events.
	Carta *et al*, 2013 [18]	A case-control study with 83 AMI patients, where a “>120 minutes” was used as the cut-off to divide the sample into “Late Responders Case Group” (43.4%) and “Early Responders Control Group” (56.6%) regarding the PH delay following AMI. Only higher alexithymia scores and contact with primary care were associated with increased PH delay. The co-presence of the two risk factors produced a cumulative risk without significant amplification.
	Meloni *et al*, 2016 [20]	A study conducted among 95 people with AMI underlined that high alexithymia was reported by 28% of the study population. Furthermore, high alexithymia, along with a non-productive coping strategy such as waiting for symptoms to go away, influenced patient responsiveness to cardiac symptoms, resulting in a significantly longer delay seeking help.
	Kenyon *et al*, 1991 [31]	An observational study pointed out that reduced awareness of bodily sensations and emotions, typical of alexithymia, significantly predicted longer PH delay in 103 AMI patients. A “>4 hours” cut-off was used for descriptive statistics, showing that 46% of 31 patients reached the hospital within 4 hours. Longer interval time was not significantly associated with demographic or medical history categories or with Type A behavior.
	Theisen *et al*, 1995 [32]	A study compared 40 patients who sought treatment for a documented AMI with 30 patients who were found on routine electrocardiogram to have had an AMI for which they did not seek medical care. Patients with unrecognized AMI showed greater alexithymia. The Authors hypothesized that, among other factors, alexithymia may impede AMI recognition and may inhibit treatment-seeking.
	O’Carroll *et al*, 2001 [33]	In a case-control study, 72 patients with AMI were divided into two groups: “delayers” (who waited more than 4 h before seeking help since experiencing their first chest pain) and “prompt attenders”. Findings showed that there was no significant difference in alexithymia levels between “prompt attenders” and “delayers”, although they tended to be higher among “delayers”.
	Sancassiani *et al*, 2021 [21]	A systematic review including 36 studies, involving 10.389 patients with an ongoing AMI found that wrong appraisal, interpretation and causal beliefs about symptoms, denial of the severity of the symptoms and high levels of alexithymia were found related to longer PH delay or PD delay.
	Preti *et al*, 2013 [19]	Out of 29 studies investigating the role of psychological factors in PH delay after AMI, 3 studies specifically assessed alexithymia, involving 258 patients. Meta-analysis of data showed that the patients with higher emotional awareness (*i.e*., low alexithymia) had a shorter PH delay after AMI.
***Topic 2 - How do assess alexithymia?***	Bagby *et al*, 1994a [35]	Addressing shortcomings of the self-report Toronto Alexithymia Scale (TAS), two studies were conducted to reconstruct the item domain of the scale. The first study resulted in the development of a new twenty-item version of the scale, the “TAS-20”, with good internal consistency and test-retest reliability, and a three-factor structure theoretically congruent with the alexithymia construct. The stability and replicability of this three-factor structure were demonstrated in the second study with both clinical and nonclinical populations by the use of confirmatory factor analysis.
	Bagby *et al*, 1994b [36]	A study that further evaluated the construct validity of the TAS-20 by examining its relationship with measures of personality traits theoretically related or unrelated to the alexithymia construct, as well as its relationship with an observer-rated measure of alexithymia in samples of university students and in a sample of behavioral medicine outpatients.
	Bagby *et al*, 2020 [37]	A review of the psychometric literature evaluated the reliability and validity of the TAS-20 and examined some of the controversies surrounding the scale and the construct it assesses. Based on the accumulated empirical evidence of 25 years, including findings from confirmatory factor analytic and convergent and discriminant validity studies, the Authors concluded that the TAS-20 is a reliable and valid instrument and accurately reflects and measures the construct.
	Taylor *et al*, 1988 [38]	The criterion validity of the Toronto Alexithymia Scale [TAS) was assessed by administering the scale to 46 patients referred to a behavioral medicine outpatient clinic. Clinical ratings derived from observed interviews served as the criterion. TAS scores were significantly higher for the group of patients identified by two out of three raters as "alexithymic" than for the group identified as "non-alexithymic." On the basis of these findings, preliminary TAS cutoff scores were suggested.
	Sifneos, 1973 [1]	The article describes the construction and the first administration of the Beth Israel Hospital Questionnaire (BIQ) to 25 psychosomatic patients (people suffering from diseases such as ulcerative colitis, asthma, peptic ulcer, rheumatoid arthritis) compared with 25 controls (people with psychiatric complaints such diagnoses as borderline personality, depression, hysterical personality, alcoholism). It is the first instrument proposed to assess alexithymia. Out of the 17 questions administered by a clinician, there are 8 key ones that attempt to sort out the ‘alexithymic’ characteristics. Thus, questions 1, 7, 8, 12, 13 and 16 must be answered ‘yes’, and questions 2 and 6 must be answered ‘no’ for the patient to be considered ‘alexithymic’.
	Bagby *et al*, 2006 [41]	The study describes the development of a valid and reliable structured interview for assessing alexithymia. After pilot testing on 136 community participants and 97 psychiatric outpatients and a series of item and scale analyses, the number of constructed questions was reduced from 60 to 24. Principal component analysis and confirmatory factor analysis revealed preliminary evidence of a hierarchical, four-factor structure, with four lower factors nested within two higher-order latent factors. This structural configuration resulted in the Toronto Structured Interview for Alexithymia (TSIA) with two domain scales and four facet scales. The TSIA and its six scales demonstrated acceptable levels of interrater, internal, and retest reliability, and correlated modestly but significantly with the 20-item Toronto Alexithymia Scale and its three-factor scales, providing some support for the concurrent validity of this interview.
	Sekely *et al*, 2018 [42]	The study used Item Response Theory (IRT) methods to analyze data from a large heterogeneous multilanguage sample (N=842) to investigate whether a subset of items could be selected to create a short version of the Toronto Structured Interview for Alexithymia (TSIA). A series of statics allowed to reduce items from 24 to 12 and to establish that, despite this reduction, 65.22% of the information was retained.
	Porcelli *et al*, 2013 [44]	The study is about the use of the Diagnostic Criteria for Psychosomatic Research (DCPR) for characterizing alexithymia in a large and heterogeneous medical population of 1190 people, in conjunction with the Diagnostic and Statistical Manual of Mental Disorders, Fourth Edition (DSM-IV) and other DCPR criteria. The item of the DCPR structured interview for alexithymia, as well as the keys for scoring, were also described.
	Galeazzi *et al*, 2004 [45]	The study aims to evaluate interrater reliability, the distribution of DCPR syndromes, and their relationship with ICD-10 diagnostic categories. 100 consecutive patients who were referred for psychiatric consultation in a university general hospital consented to assessment for DCPR syndromes as elicited in a joint interview conducted by two researchers. The results showed excellent interrater agreement. DCPR criteria appear to be a useful, reliable, and promising approach in the assessment and description of psychological distress in medical patients, particularly for demoralization, alexithymia, illness denial, and type A behavior.
	Porcelli & De Carne, 2001 [46]	The main aim of the study was to investigate the criterion-related validity of the DCPR for alexithymia (DCPR-A). The sensitivity of the DCPR-A together with the TAS-20 was 70.2%, specificity was 81.6%, positive predictive power 88.9%, negative predictive power 66.0% and overall hit rate 46.8%. DCPR-A positives scored significantly higher than DCPR-A negatives on the TAS-20 scores.
	Bagby *et al*, 2021 [47]	The study describes the development and validation of the “informant form” version of the TAS-20, the 20-item Toronto Alexithymia Scale – Informant Form (TAS-20-IF), to address and overcome the concern about the individuals’ ability to accurately self-report difficulties identifying and describing feelings if they are deficient in those abilities. The psychometric properties (items and scales) of TAS-20 and TAS-20-IF were tested among a large sample of “target” and “informants” nominated by the “targets”. These properties were adequate, and the three-factor structure of the TAS-20-IF was supported; the correlation between the two versions was statistically significant and the factor structures were similar.
***Topic 3 - Treating alexithymia***	Ogrodniczuk *et al*, 2011 [48]	A review article summarized findings from a series of studies that examined the effect of alexithymia on various aspects of the psychotherapeutic enterprise. Findings indicated that alexithymia has little effect on patients' treatment preferences, yet there was some tendency for alexithymic patients to prefer group therapy. However, alexithymia was associated with poor outcome in both traditional psychodynamic psychotherapy and supportive therapy. This negative effect was found in individual and group psychotherapies. In the context of group therapy, higher levels of alexithymic features elicited negative reactions from one's therapist, which partially contributed to the poor outcome experienced by such patients.
	Douquette, 2020 [49]	A theoretic article with the purpose to propose a new clinical access point for the evaluation and treatment of the deficits in emotional awareness demonstrated in alexithymia. Three clinical cases were presented to illustrate considerations of alexithymic etiology and treatment salient to the clinicians, in order to support their understanding of patients against the backdrop of current psychodynamic and neuroscientific research, which will thereby increase treatment options and benefits. The implications of the historical, psychological, and somatic aspects of patients’ experience were considered, to better understand their diminished ability to: experience and represent emotional experience as distinct feeling states; signify the relevant meaning of affective experience; and incorporate such with cognitions to adaptively guide behavior.
	Beresnevaite, 1995 [50]	A preliminary controlled trial involved 20 post-myocardial infarction (MI) patients were placed in a treatment group, which received weekly group psychotherapy for 4 months. 17 post-MI patients were placed in a comparison group which received two educational sessions over a period of 1 month. All subjects completed the Toronto Alexithymia Scale (TAS) before the start of group therapy, at the end of the 4-month period, and in follow-up assessment after 6-month, 1-year, and 2-year intervals. In the psychotherapy treatment group, there was a significant reduction in the mean TAS score following group therapy, which was maintained over the 2-year follow-up period. In the educational group, there were no significant changes in mean TAS scores between the initial testing and any of the follow-up intervals. Over the 2-year follow-up period, patients with decreased alexithymia following group therapy experienced fewer cardiac events than patients whose alexithymia remained unchanged.

**Note:** CVD: cardiovascular disease; AMI: acute myocardial infarction; STEMI: ST-segment elevation myocardial infarction; CHD: coronary heart disease; PH delay: pre-hospital delay; PD delay: patient-decisional delay; DCPR: Diagnostic Criteria for Psychosomatic Research

## Data Availability

The data supporting the findings of the article is available in the Zenodo Repository.

## LIST OF ABBREVIATIONS

TSIA: = Toronto structured interview for alexithymia
DIF: = Difficulty identifying feelings
DDF: = Difficulty describing feelings
EOT: = Externally oriented style of thinking
IMP: = Imaginal processes
